# Unusual papillary thyroid carcinoma with hyalinizing trabecular tumor-like feature in a young female patient: a case report

**DOI:** 10.1186/s13256-023-03870-9

**Published:** 2023-03-28

**Authors:** Yoshiko Umekita, Kazumi Umeki, Fumiaki Kawano, Hiroyuki Tanaka, Hiroaki Kataoka

**Affiliations:** 1 Division of Oncopathology and Regenerative Biology, Department of Pathology, Faculty of Medicine, University of Miyazaki, 5200 Kihara, Kiyotake, Miyazaki, 889-1692 Japan; 2 Department of Medical Life Sciences, School of Medical Life Sciences, Kyushu University of Health and Welfare, Yoshino-Cho 1714-1, Nobeoka, Miyazaki, 882-8508 Japan; 3 Division of Gastrointestinal, Endocrine and Pediatric Surgery, Department of Surgery, Faculty of Medicine, University of Miyazaki, Miyazaki, Japan

**Keywords:** Hyalinizing trabecular tumor, Papillary thyroid carcinoma, Basement membrane materials, Fine-needle aspiration cytology, Case report

## Abstract

**Background:**

Hyalinizing trabecular tumor is a rare follicular cell-derived thyroid neoplasm that is considered to be a borderline tumor with malignant potential rather than a benign tumor. The detection of *RET/PTC* rearrangements and nuclear cytologic features suggests a relationship between hyalinizing trabecular tumor and papillary thyroid carcinoma. Some recent observations of pathogenic genetic alterations in hyalinizing trabecular tumor have indicated that hyalinizing trabecular tumor is not related to papillary thyroid carcinoma, and should be considered an independent entity. Here we present a case of papillary thyroid carcinoma with hyalinizing trabecular tumor-like features and discuss its interesting aspects and diagnostic issues from a histopathological perspective.

**Case presentation:**

A 19-year-old Japanese woman with an enlarged thyroid gland was admitted to our hospital. Based on fine-needle aspiration cytology, the enlarged nodule was suspected to be a follicular lesion or follicular tumor. A nodular lesion approximately 3 cm in diameter was detected in the left lobe of the thyroid gland. Histological analysis revealed that the tumor cells were mainly arranged in follicles. Solid nests with occasional trabecular arrangements and papillary structures were intermingled, and the tumor cells showed ground-glass nuclei and occasional nuclear grooving. Petaloid and block-like periodic-acid-Schiff and periodic-acid-methenamine-positive basement membrane components were observed in the interstitium of the solid portions of the tumor. Incomplete membranous immunoreactivity of MIB-1 (Ki-67 (cell prolferation marker)) was also observed in the cells within the solid areas. Moreover, this tumor displayed extracapsular invasion and metastasis to the perithyroidal lymph nodes, suggesting that it may be a malignant tumor. However, *BRAF*^*V600E*^ mutation, *RET/PTC* rearrangements, and *PAX8/GLIS 1* and *PAX8/GLIS 3* rearrangements were not detected.

**Conclusion:**

We diagnosed the tumor as a papillary thyroid carcinoma with characteristic features of hyalinizing trabecular tumor. Importantly, this case may indicate a possible relationship between papillary thyroid carcinoma and hyalinizing trabecular tumor, although specific genetic alterations could not be detected. We also discuss the preoperative diagnostic difficulties with fine-needle aspiration cytology and the unusual pathological findings in this case.

## Background

Papillary thyroid carcinomas (PTCs) and hyalinizing trabecular tumors (HTTs) are thyroid follicular cell-derived neoplasms. PTC is the most common malignant thyroid malignancy, whereas HTT is rare. HTTs were first described in 1987 by Carney *et al*. as benign hyalinizing trabecular adenomas [1]. However, it is still unclear whether HTTs are benign or malignant, as invasion and metastasis have been reported in some cases [1]. Additionally, a relationship with PTC has been proposed based on the detection of *RET* gene rearrangements in approximately half of HTTs [1]. In contrast, Nikiforva *et al*. reported that all HTT cases were positive for *PAX8-GLIS3* or *PAX8-GLIS1* fusion genes, but negative for *RET/PTC* fusion genes [2].

Here we report an unusual case of a thyroid tumor that showed predominantly follicular growth, along with scattered solid and trabecular nests with basement membrane material and papillary growth. Repeated preoperative cytological examination failed to detect PTC. After surgery, we diagnosed the patient with PTC with HTT-like features. In this case report, we discuss the possible relationship between PTC and HTT-like lesions. We also discuss the limitations of the preoperative cytological diagnosis encountered in this case.

## Case presentation

**Fig. 1:**
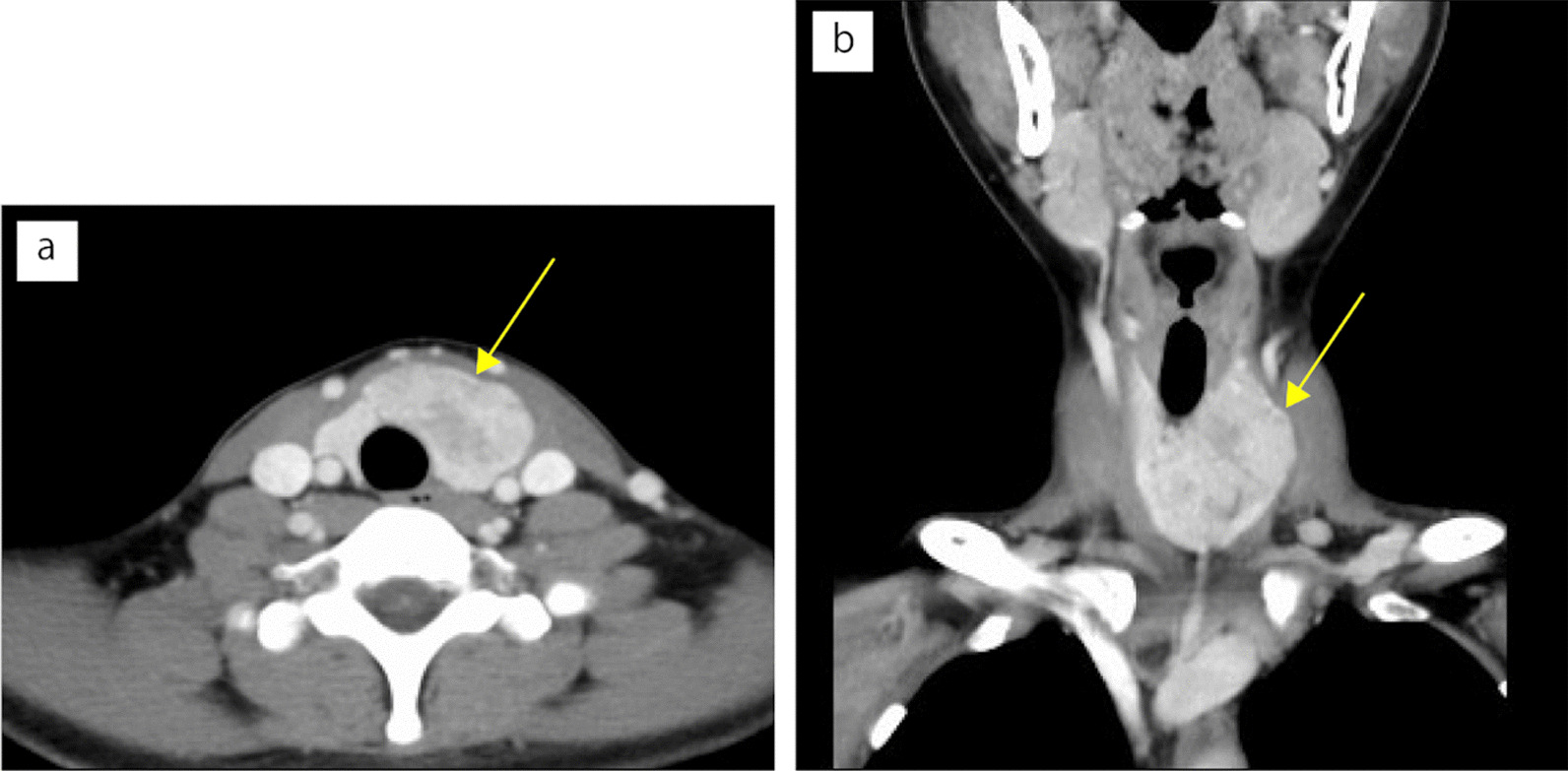
Contrast-enhanced computed tomography. **a**, **b** A nodular lesion (40 × 48 mm) is observed in lower pole to isthmus of the thyroid left lobe. The lesion showed heterogeneous enhancement. The arrows indicate thyroid tumor

**Fig. 2:**
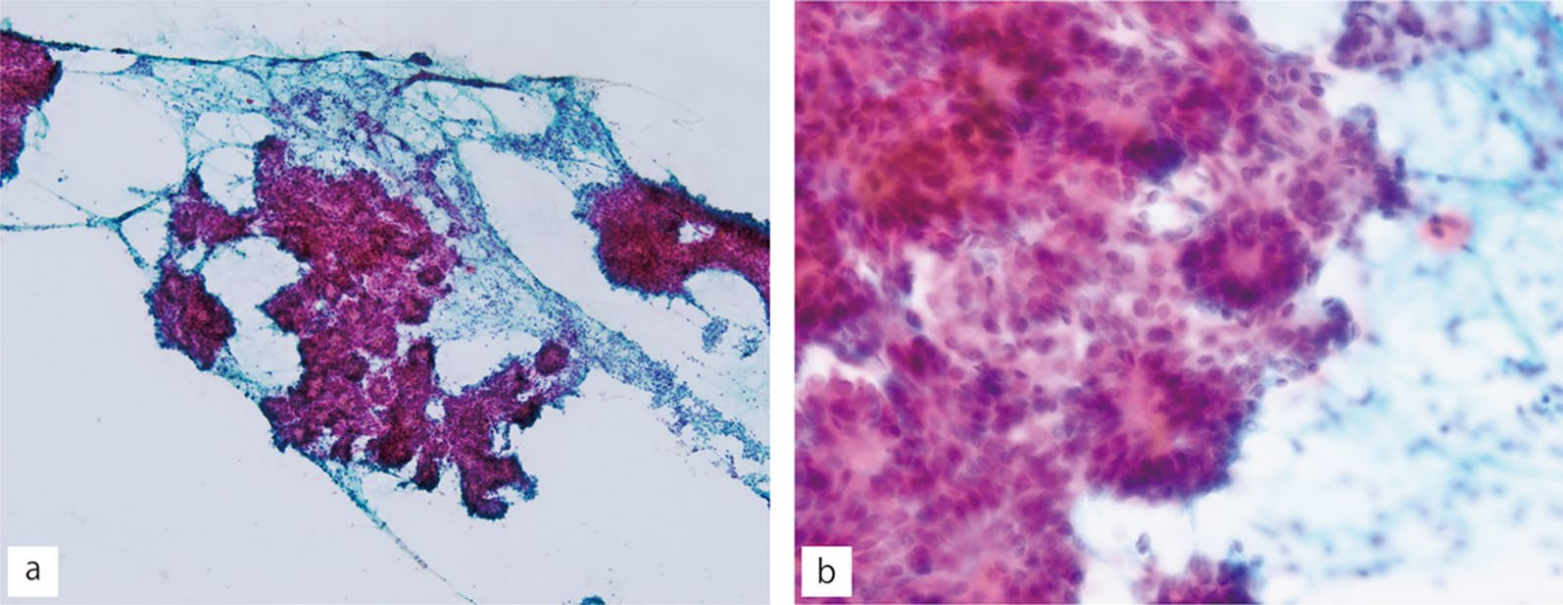
Papanicolaou stain of fine-needle aspirate specimen. **a** Low-power magnification (×100) showed various features of follicular cell aggregation (for example, follicular-, papillary-, and sheet-like). **b** High-power magnification (×400) showed relatively monotonous nuclei. Nuclear grooving and inclusion are unclear

A 19-year-old Japanese woman had presented with an enlarged thyroid gland of the left lobe at the age of 14 years during a regular medical check-up at school. Following this observation, the patient was closely monitored without any treatment. The nodular lesion gradually became visible and enlarged. Fine-needle aspiration cytology (FNAC) examinations were performed thrice, and the tumor was diagnosed as benign. Five years after the initial detection of thyroid enlargement, the lesion reached a size of 45 × 40 mm and revealed heterogeneous enhancement on contrast-enhanced computed tomography (CT) images (Fig. 1a, b). Her serum thyroglobulin level was high at 1220 μg/L, while her thyroid hormone levels remained within the normal range. The fourth FNAC revealed numerous epithelial cells arranged in follicle-, papillary-, and sheet-like patterns. The cells were round and nearly uniform, suggesting neoplastic etiology. There were no nuclear findings suggestive of papillary carcinomas in the nuclei, such as nuclear grooves or nuclear inclusion bodies (Fig. 2a, b). Subsequently, the patient was judged to have a relative indication for surgery, left hemithyroidectomy using video-associated neck surgery (VANS) method. The resected thyroid tissue was submitted to the pathology department after formalin fixation.

**Fig. 3:**
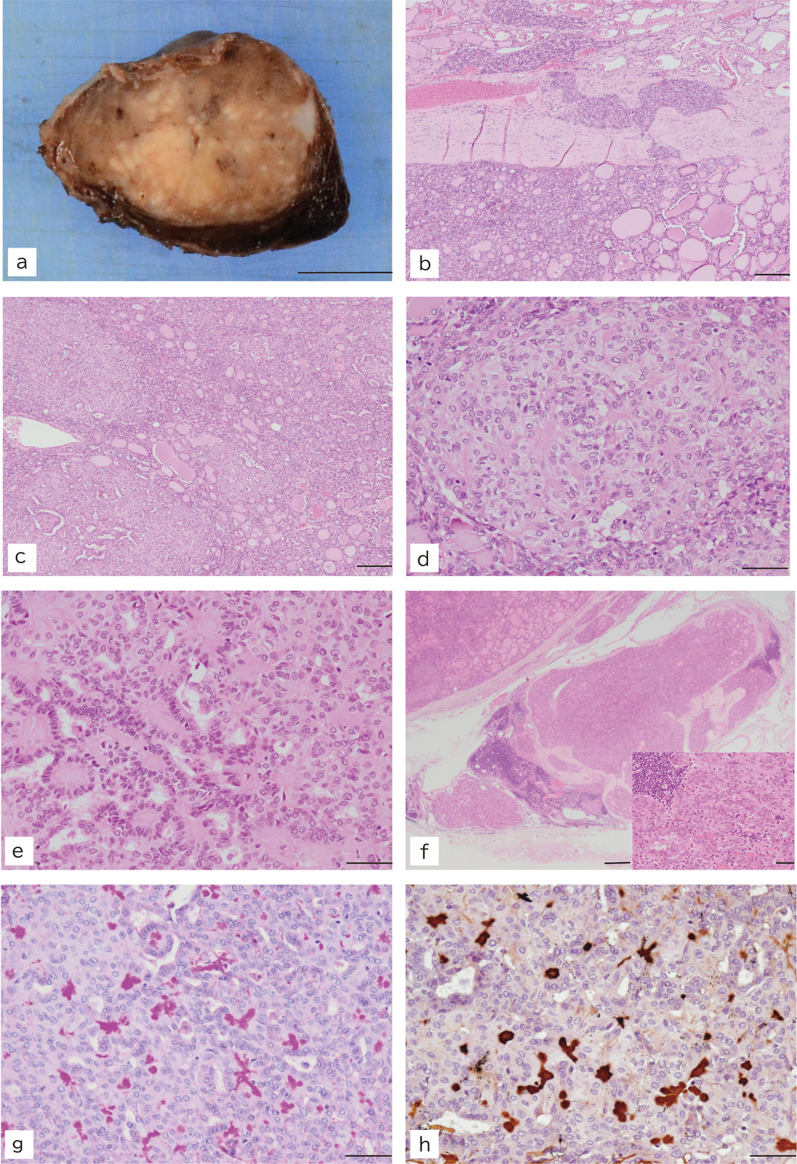
Macroscopic and histological findings. **a** Cut surface of the thyroid tumor (bar, 10 mm). **b** The tumor invaded the surrounding thyroid tissue. Hematoxylin and eosin staining (bar, 200 μm). **c** The tumor exhibited various growth pattern; follicular, solid, and papillary (hematoxylin and eosin; bar, 200 μm). **d** Solid growth portion associated with vague trabecular arrangement. Block-like or petaloid eosinophilic deposition was observed in the interstitium (hematoxylin and eosin; bar, 50 μm). **e** The papillary structure was adjacent to or mixed in with the solid portion. Eosinophilic depositions were also found in papillary cores (hematoxylin and eosin; bar, 50 μm). **f** Metastatic lesions of the regional lymph nodes. A higher magnification image of the metastatic tumor cells is shown in the inset (hematoxylin and eosin; bars, 500 μm and 20 μm for low- and high-magnification images, respectively). **g **PAS staining. **h** Periodic acid-methenamine-silver staining. The deposits were positive for basement membrane materials following periodic acid-Schiff and periodic acid-methenamine-silver staining. Bars, 50 μm

The cut surface of the tumor showed that it was well-demarcated; however, it exhibited irregular contours with a thin incomplete capsule as well as heterogeneous white/light tan-colored areas upon formalin fixation. The diameter of the excised tumor mass was 3.1 × 2.3 cm (Fig. 3a).

Microscopically, the tumor displayed various distinct growth patterns: follicular, solid, trabecular, and papillary. The tumor cells invaded the surrounding thyroid tissue and breached the fibrous capsule (Fig. 3b). A follicular growth pattern with colloid was predominant, where the tumor cells had mildly enlarged nuclei but lacked the typical nuclear features of papillary carcinoma. In contrast, several foci of solid nests with interlacing trabeculae composed of elongated cells were present, and the papillary structures were associated with or adjacent to the solid nests (Fig. 3c). Moreover, block-like or petaloid eosinophilic depositions were observed in the interstitium of the solid nests, and occasionally in the papillary cores (Fig. 3d, e). The tumor cells of the solid and papillary portions showed the characteristic nuclear features of PTC, such as ground-glass appearance, nuclear grooving, and nuclear contour irregularity (Fig. 3e). Regional lymph node metastasis was also detected, and both follicular and solid architectures were concomitantly observed in the metastatic lesions (Fig. 3f). The eosinophilic deposits around the tumor nests were positive for periodic acid-Schiff (PAS) and periodic acid-methenamine-silver (PAM) staining, consistent with basement membrane material staining (Fig. 3g, h).

**Fig. 4:**
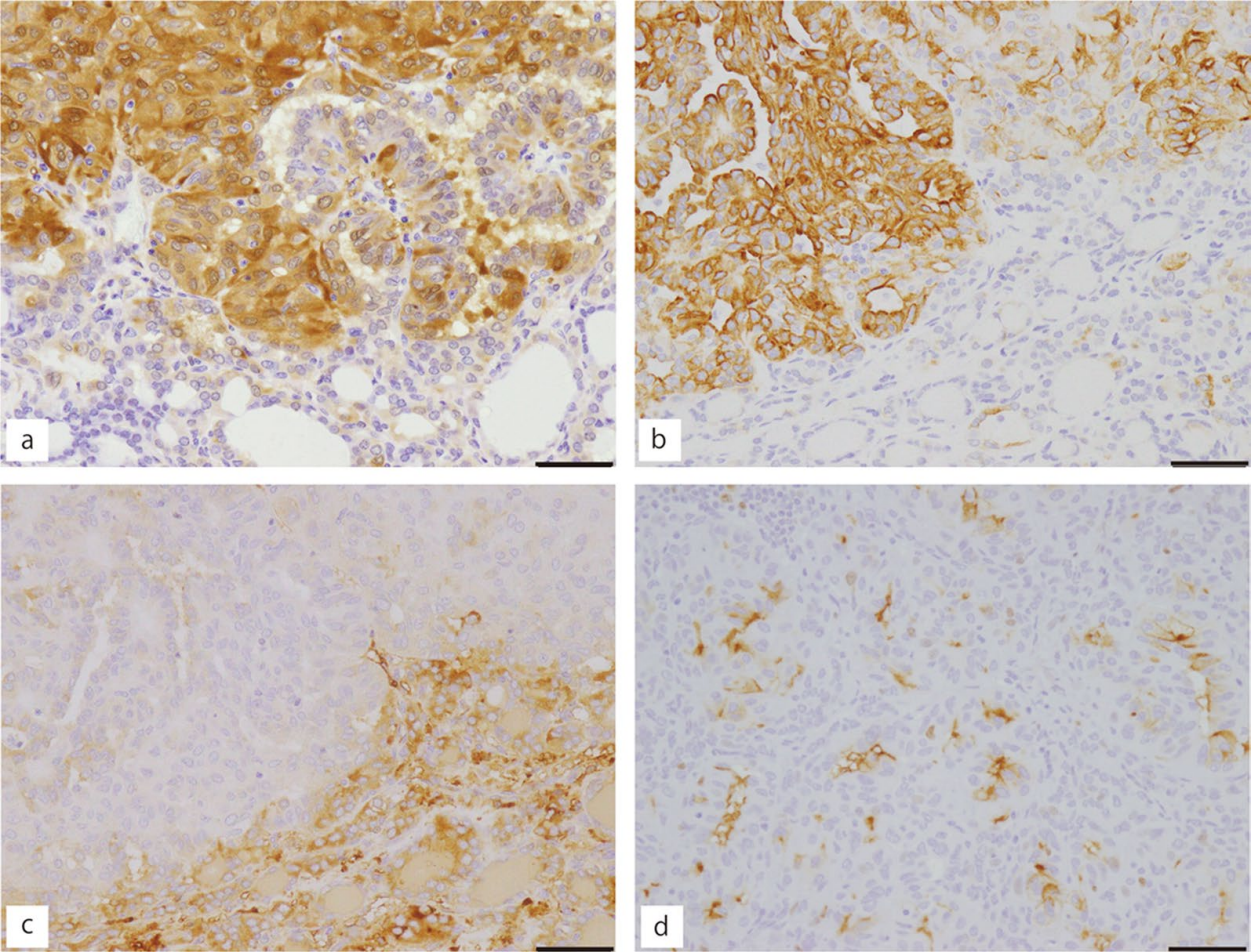
Immunohistochemical analysis for **a** Cytokeratin19, **b** galectin 3, **c** thyroglobulin, and **d** MIB-1. The solid/papillary portion is observed in the upper half and the follicular portion is in the lower half. Various degrees and distributions of the staining were found (**a**–**c**). MIB-1 staining of the papillary/solid portion showed incomplete and weak membranous staining in the tumor cells (**d**). Bars, 50 μm

Immunohistochemical analysis revealed that the tumor cells were diffusely positive for thyroid transcription factor 1 (TTF-1) and galectin 3, with more intense galectin 3 immunoreactivity in the solid/trabecular and papillary portions (Fig. 4a). Cells in the solid/trabecular and papillary portions were positive for CK19 and 34βE12, whereas those in the follicular structures were negative (Fig. 4b). In contrast, the follicular area was strongly positive for thyroglobulin (Tg), but only faint signals were observed in the solid, trabecular, and papillary portions (Fig. 4c). MIB-1 (Ki-67 (cell prolferation marker)) staining revealed an incomplete and weak membranous staining pattern in the solid and trabecular portions (Fig. 4d). Table 1 summarizes the results of immunohistochemical analysis. Point mutations in *BRAF*^*V600E*^ were not detected using polymerase chain reaction (PCR) followed by direct sequencing [3]. *RET-PTC1*, *RET-PTC2, RET-PTC3*, *PAX8-GLIS1,* as well as *PAX8-GLIS3* rearrangements were not detected by RT–PCR [2, 4, 5]. *KRAS* (codons 12,13,59,61,117, and 146) and *NRAS* (codons 12, 13, 59, 61, 117, and 146) gene mutations were also not detected (performed at the laboratory of SRL Inc., Tokyo, Japan). Collectively, the final pathologic diagnosis was PTC with HTT-like features.

**Table 1:** Immunoreactivity by pathological findings

Antibody	HTT-like portion	Papillary portion	Follicular portion
TTF-1	+	+	+
Tg	− ~ +	− ~ +	+
CK19	+	+	−
34βE12	+	+	−
Galectin-3	+	+	−
MIB-1	+ , membrane	− ~ + , membrane	−

HTT: Hyalinizing trabecular tumor; TTF-1: Thyroid transcription factor-1; Tg: Throglobulin; CK19: Cytokeratin 19; MIB-1: Ki-67 (cell proliferation marker)

A complete physical examination was performed after surgery. Cervical ultrasonography showed enlarged lymph nodes of levels II and IV. Positron emission tomography–computed tomography (PET–CT) revealed small nodules in the lower lobes of both lungs, suggesting the presence of pulmonary metastasis. Subsequently, additional resections of the right lobe of the thyroid and the regional lymph nodes were performed. Metastasis was found in the left lymph nodes at level IV, although no definite cancer was detected in the right lobe of the thyroid gland. According to the eight edition of the Union for International Cancer Control (UICC 8th ed.), final staging of this case was considered pT2, pN1b, cM1, Stage II. After the second surgery, the patient underwent two rounds of radioactive iodine therapy with monitoring of thyroglobulin levels. The pulmonary lesions disappeared in the images. Currently, about 5 years have passed since the surgery, and no apparent recurrence has been observed.

## Discussion and conclusion

PTC is the most common malignant tumor of the thyroid gland [1]. In contrast, HTT is a rare follicular-derived neoplasm that exhibits a trabecular growth pattern of elongated or polygonal large cells associated with extracellular basement membrane material deposition [1]. Herein, we report a case of PTC with HTT-like features. Several cases of PTC combined with HTT have been reported [6–8, 10]. Among them, the case reported by González *et al*. particularly resembles our case. They reported a case of a 19-year-old woman with a portion of PTC with HTT-like morphology within a follicular adenoma [6].

There were two noteworthy points in this case. First, there was a discrepancy between the preoperative cytological diagnosis and the postoperative histopathological diagnosis. The patient underwent FNAC four times and was diagnosed with a benign lesion in the first three examinations and a suspected follicular tumor in the last examination; she had never been diagnosed with papillary carcinoma preoperatively. The resected thyroid lesion was composed predominantly of tumor cells arranged in a follicular growth pattern with intraluminal colloid, and HTT-like areas with basement membrane material deposits and papillary growth areas with typical nuclear features of PTC were only scattered. We assume that this histopathological pattern resulted in an inaccurate preoperative cytological diagnosis. However, in the above-mentioned case reported by González *et al*. [6], which was similar to our case, the lesion was diagnosed as a follicular tumor based on preoperative cytology.

Second, typical PTC areas were almost invariably observed adjacent to HTT-like areas. This suggests a close relationship between PTC development and HTT. Although most HTTs are benign, rare cases of invasion or recurrence have been reported [1]. The detection of *RET/PTC* gene rearrangements, which are observed in PTCs, in approximately half of HTTs has led to a debate as to whether HTTs are independent diseases or subtypes of papillary carcinoma with favorable prognosis [8, 9]. Recently, Nikiforva *et al*. reported the presence of the fusion genes *PAX8-GLIS3* and *PAX8-GLIS1* in all HTTs [2]. In contrast, none of the HTTs had *RET/PTC* gene rearrangement characteristics of PTC, and all 220 cases of PTC were negative for *PAX8-GLIS3* rearrangement, although *PAX8-GLIS1* fusion was detected in one case. These authors proposed that HTT is an independent disease that is distinct from PTC. In the present case, we did not detect any *PAX8/GLIS* rearrangement or other mutations in PTC, such as *BRAF*^*V600E*^, *RAS*, or any *RET/PTC* rearrangements. However, considering the presence of a papillary growth portion and the observation of invasive growth with lymph node metastasis, the tumor was diagnosed as PTC with HTT-like features. HTTs and PTCs have many histological similarities, and there have been several case reports of PTC and HTT-like growth forms adjacent to and intermingled within tumors [6–8, 10]. As observed in our case, we suggest that PTC and HTT may be on the same spectrum in tumorigenesis.

## Data Availability

Not applicable to this article.

## Abbreviations

FNAC: Fine needle aspiration cytology
*BRAF*: V-raf murine sarcoma viral oncogene homolog B1
*RET*: Receptor tyrosine kinase
PAX8: Paired box gene 8
GLIS1: Glis family zinc finger 1
GLIS3: Glis family zinc finger 3
PTC: Papillary thyroid carcinoma
HTT: Hyalinizing trabecular tumor
PAS: Periodic acid-Schiff
PAM: Periodic acid-methenamine-silver
TTF-1: Thyroid transcription factor-1
PCR: Polymerase chain reaction
KRAS: Kirsten RAS
NRAS: Neuroblastoma RAS
CK: Cytokeratin
Tg: Thyroglobulin
PET–CT: Positron emission tomography–computed tomography
